# Supraventricular Tachycardias Using Multiple Accessory Pathways

**DOI:** 10.1002/joa3.70276

**Published:** 2026-01-26

**Authors:** Takumi Yamada

**Affiliations:** ^1^ UF Health Cardiovascular Center University of Florida College of Medicine Jacksonville Florida USA

**Keywords:** accessory pathway, catheter ablation, multiple, supraventricular tachycardia

## Abstract

Three atrioventricular reciprocating tachycardias (AVRTs) using 3 accessory pathways (APs) occurred with the His bundle eliminated by the previous ablation. Two AVRTs using 2 right APs rotated reversely, and the other AVRT was one with a retrograde conduction through the left AP and anterograde conduction through the 2 right APs.

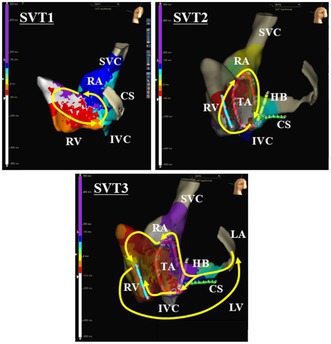

The incidence of multiple accessory pathways (APs) has been reported at 3%–18% during the radiofrequency ablation era [1]. Multiple APs present multiple electrophysiological challenges in their diagnosis and treatment.

A 27 year old woman from South America with a past medical history of WPW syndrome and wide QRS complex tachycardia was referred for catheter ablation. She had undergone catheter ablation of supraventricular tachycardia (SVT) in her country at the age of 10, but no information about the procedure was available. She presented at the emergency department with pre‐syncope and tachycardia. She was found to have a wide QRS complex tachycardia with a rate of 200 beats per minute. A high dose of adenosine was administered intravenously with no interruption of the tachycardia and electrical cardioversion was required to terminate the tachycardia. Echocardiography revealed no evidence of Ebstein's anomaly. Written, informed consent was obtained, and an electrophysiological study was performed. At baseline, 12‐lead electrocardiograms exhibited sinus rhythm with preexcitation (Figure 1). Multipolar mapping catheters were positioned in the coronary sinus, His bundle region, and right ventricle, and along the tricuspid annulus (TA) (Figure 2). Activation mapping was performed with an Advisor HD Grid Mapping Catheter (Abbott Laboratories, Abbott Park, IL, USA). It was noted that His bundle electrograms were not recorded during meticulous mapping at both the right and left ventricular septum. Intravenous administration of adenosine during sinus rhythm never altered the QRS morphologies. Activation mapping during sinus rhythm and pacing from the right ventricle revealed 2 APs with both anterograde and retrograde conduction at anterior and posterior aspects of the TA (Figure 3A). Atrial extrastimulation revealed that the effective refractory periods of both APs were the same (290 msec). Three different SVTs exhibiting wide QRS complexes were induced by atrial pacing with an intravenous infusion of isoproterenol. SVT1 exhibited left bundle branch block and left inferior axis QRS morphology with a tachycardia cycle length of 235 msec, SVT2 left bundle branch block and left superior axis QRS morphology with a tachycardia cycle length of 243 msec, and SVT3 left bundle branch block and left superior axis QRS morphology with a tachycardia cycle length of 267 msec (Figure 3B–D). Activation mapping during the tachycardias revealed that SVT1 was an atrioventricular reciprocating tachycardia (AVRT) with anterograde conduction through the right anterior (12 o'clock) AP and retrograde conduction through the right posterior (6 o'clock) AP, SVT2 was an AVRT with retrograde conduction through the right anterior AP and anterograde conduction through the right posterior AP, and SVT3 was an AVRT with retrograde conduction through the left lateral (3 o'clock) AP and anterograde conduction through the right anterior and posterior APs (Figure 3B–D). Radiofrequency catheter ablation was performed at the posterior aspect of the TA and left lateral aspect of the mitral annulus, and the 2 APs were successfully eliminated (Figure 2). After the ablation, 12‐lead electrocardiograms during sinus rhythm exhibited preexcitation (Figure 1). Mapping around the anterior aspect of the TA caused a transient atrioventricular block. Intravenous administration of adenosine during sinus rhythm never altered the QRS morphologies. These findings suggested that the His bundle had been ablated by the previous ablation. Atrial extrastimulation revealed that the effective refractory period of the right anterior AP was 290 msec. Considering the patient's age and low risk of sudden cardiac death associated with the remaining AP, catheter ablation of the AP was not performed.

**FIGURE 1:**
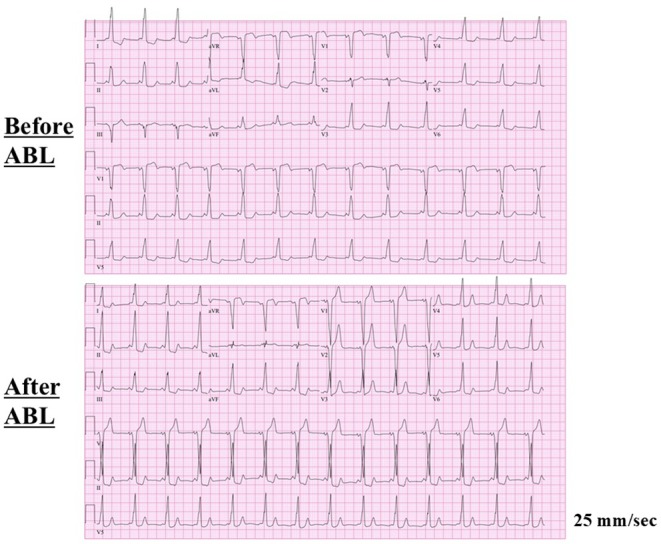
Twelve‐lead electrocardiograms before and after the ablation (ABL).

**FIGURE 2:**
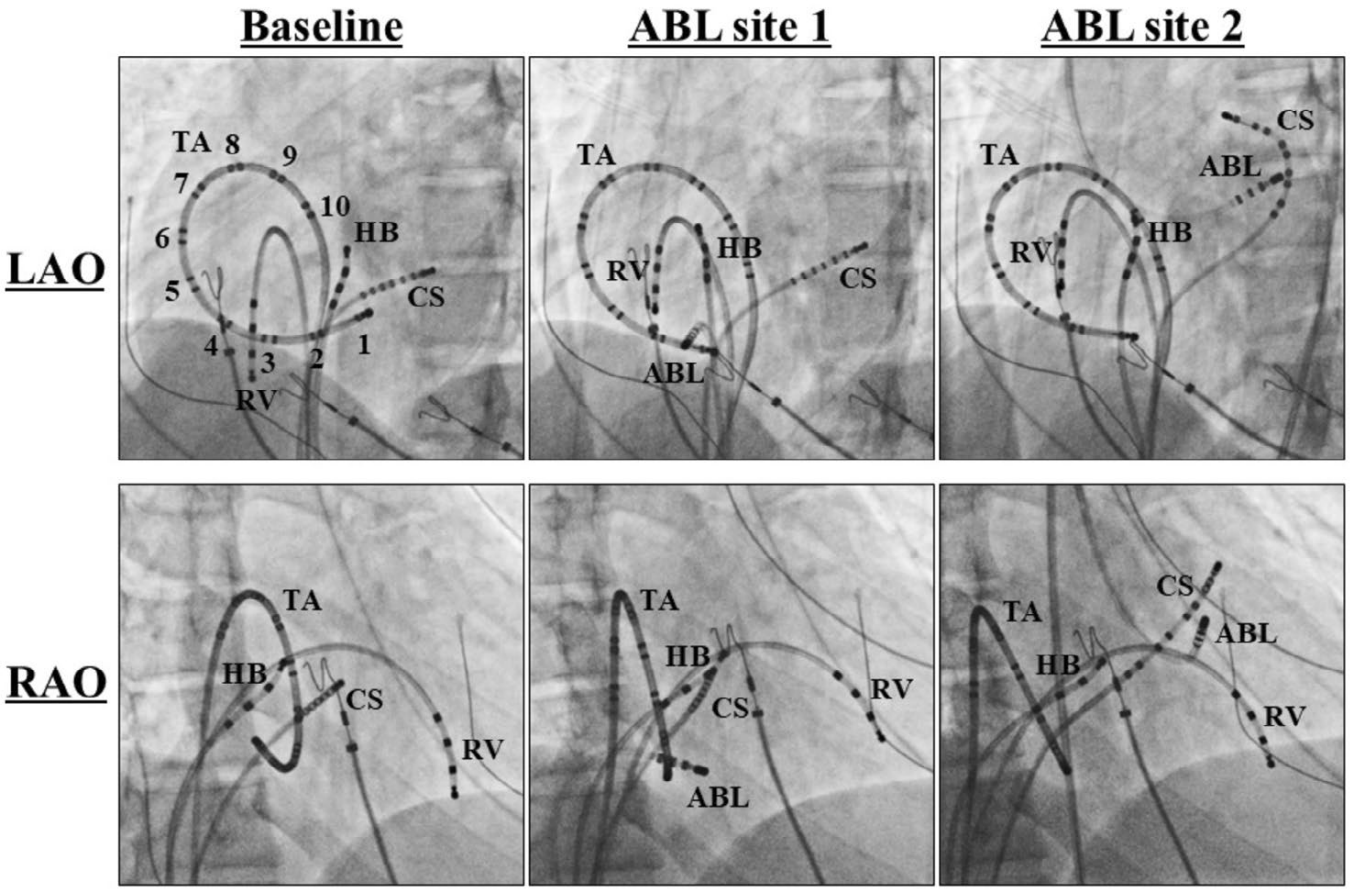
Fluoroscopic images exhibiting the catheter positions at baseline (left panels), and the successful ablation (ABL) sites at the posterior aspect of the tricuspid annulus (middle panels) and lateral aspect of the mitral annulus (right panels). CS, coronary sinus; HB, His bundle; LAO, left anterior oblique projection; RAO, right anterior oblique projection; RV, right ventricle; TA, tricuspid annulus.

**FIGURE 3:**
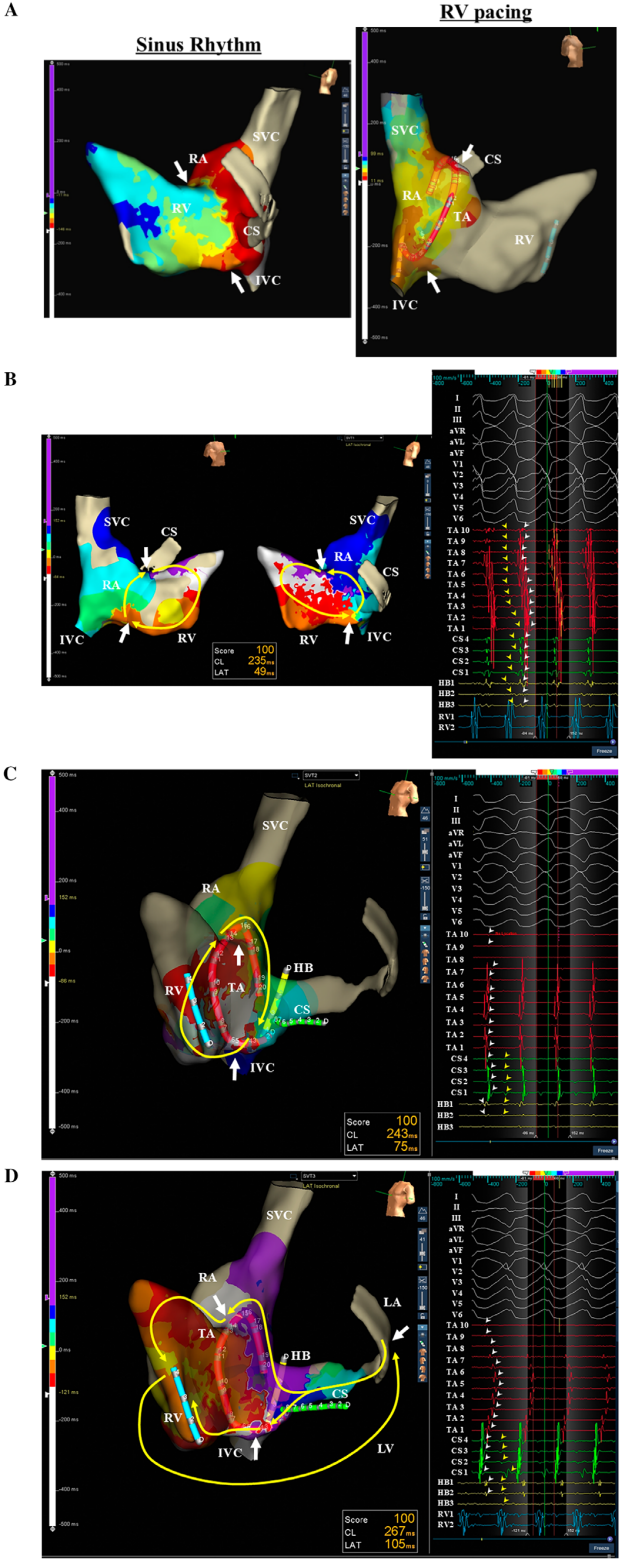
(A) Activation maps recorded during sinus rhythm (left panel) and RV pacing (right panel) at baseline. (B) Activation maps and cardiac tracings recorded during supraventricular tachycardia (SVT)1. (C) Activation map and cardiac tracings recorded during SVT2. (D) Activation map and cardiac tracings recorded during SVT3. The yellow and white arrows indicate the reentrant circuits of the SVTs and locations of the accessory pathways, respectively. The yellow and white arrowheads indicate the ventricular and atrial potentials, respectively. IVC, inferior vena cava; LA, left atrium; LV, left ventricle; RA, right atrium; SVC, superior vena cava; X 1 to 10 = the first to tenth electrode pairs of the relevant catheter. The other abbreviations are as in the previous figures.

During more than 3 years of follow up, the patient has been free of any episodes of tachycardias and syncope without any antiarrhythmic drugs.

This patient was unique for several reasons. First, this patient had a total of 3 APs including 2 right and one left. Second, 3 different AVRTs using multiple APs were induced. Two of those AVRTs used the same right APs but their reentrant circuits rotated reversely. Both of those 2 APs possessed bidirectional conduction, which was required for those AVRTs to occur. AVRTs using 2 APs are clinically challenging because they are very rapid and hemodynamically compromised. In addition, they are refractory to atrioventricular nodal blocking agents. In this case, the SVTs could never be terminated by adenosine and required cardioversion for their termination. Third, the His bundle had been eliminated by the previous ablation. The patient had not had any SVTs for more than 15 years since the ablation, but it was malpractice. There could be an argument about whether the remaining AP should be ablated or not. Catheter ablation of the remaining AP should result in complete atrioventricular block, requiring a permanent pacemaker. Given that the patient was young and the risk of sudden cardiac death associated with the remaining AP was low, abandonment of catheter ablation of the remaining AP with careful long‐term follow up might have been considered clinically reasonable.

## Funding

The author has nothing to report.

## Conflicts of Interest

The author declares no conflicts of interest.

## Data Availability

The data that support the findings of this study are available on request from the corresponding author. The data are not publicly available due to privacy or ethical restrictions.
